# Editorial

**Published:** 1865-06

**Authors:** 


### Editorial.

### SANITARY COMMISSIONS AND FAIRS. THEIR MORAL AND MEDICAL RESULTS.

The great North-Western Fair for the benefit of the Sanitary Commission and of the Soldier's Home, is now in full and successful progress. As the iniquitous rebellion is now put down by the skill of our generals and the patriotic valor of our troops, helped not a little, we know, by the quiet services of the medical officers of the army, this is probably the last fair the Sanitary Commission will ever hold, until some future war shall demand new efforts: hence, it is a suitable time to review its work and judge of its usefulness.

The idea of the Commission is derived from the British society of the same character, which was established during the Crimean war. The primary object of such a body is, of course, to promote the health of the army by every possible means. When the Commission first commenced its labors, many judicious men felt grave doubts as to its usefulness. Officers of the army felt that a sort of insult was implied by the presence and labors of a body whose very existence presupposed some deficiency in the proper management and supply of the army, and especially of its medical department. Civilians asked the question, whether it was wise to attempt to supplement any deficiencies of the government, and of the army officers, in the supplying and relieving of the men by means of an outside organization, which had no authority in the service, and might become loose and injudicious in its action. It was a serious question whether it would not be better to have every proper relief and supply furnished by the government through its own compact and efficient official machinery, and, if there was any deficiency in that respect, to lash the authorities up to their duty by public opinion and the press. It was feared, also, that if the Commission proved efficient, that the government might come, in some measure, to rely on it for those sanitary helps which ought to be furnished by official authority, and so the care of these important interests be insensibly transferred from the compact, well-organized, and responsible medical depart ment to a less complete outside body, which, from the nature of the case, might prove irregular and uncontrollable in its action. Such were the grave questions which passed through the minds of thinking men at the outset of the war.

After four years' experience, these fears have happily proved unfounded, and it is desirable that a clear and philosophic view of the character and tendency of the Sanitary operations should exist among medical men.

1st. There are serious objections to the government adding to the cumbrousness of army supplies and transportion, by providing a regular system of luxuries and sanitary conveniences, in such a way as to be always accessible. It adds to the *impedimenta*, and increases the force of clerks, teamsters, quartermasters, and other non-combatants, and opens the door to many abuses. Even the simple rule for a hospital fund, has to be suspended often in long or rapid marches. At such times the army must strip itself for the fight, and carry nothing, and provide for nothing but absolute necessities. It is well, therefore, to allow the people to make voluntary contributions to the comfort of the sick and wounded, whenever the position and movements of the forces permit. It is also true that there is a certain indirect benefit to the army, in various ways, from this mode of communication with those in civil life: for, first of all, the fact that hospitals are seen and watched by the public, and liable to the praise and censure of those at home, and of the public press, is a powerful incentive to the medical officers to have everything accomplished in a creditable manner, and to cause their patients to feel comfortable and cheerful, by every possible means, in order that their reports may be of a complimentary character.

2d. The agents of the Sanitary Commission furnish an avenue through which the sympathies of the armies in the field and of the people at home mingle with and correct each other. If there ever shall be danger that the overpowering influence of a great military chief may be sufficient to lead the army on to the destruction of civil liberty, it will be well that the people have representatives fresh from their midst who will convey to the army the sentiments of those at home, and to those at home the feelings of the army, without the intervention of official dispatches. This communication being well kept up, the troops will be one in sentiment with the people, and no man can mount over them to despotic power, unless both people and soldiers agree in desiring it.

Lastly. Much valuable information is conveyed to the soldiers, officers, and surgeons, concerning their health. The documents published by the Commission at the beginning of the war, on gunshot wounds and other topics, were of great benefit to the medical officers. The final reports upon hospital and other medical matters, for which the facts are now being gathered, will also be of the utmost value in any future war. On the whole, therefore, we are agreeably impressed with the belief that the Sanitary Commission has been the instrument of great and permanent good.

---

TRANSACTIONS OF THE STATE MEDICAL SOCIETY.—Several of the papers read before the State Society have not yet been forwarded to the Publishing Committee, and the publication of the Transactions is delayed in consequence. It is hoped that these papers will be sent without delay, to Prof. N. S. DAVIS, Chicago, Ill.

The American Medical Association.—The meeting now in progress at Boston, is worthy of notice, as being the first since the war commenced which has had anything like a Southern delegation present. The war is now closed, and the objects for which it was carried on have been gloriously won. Now is the time, therefore, to turn our attention again to the culture and elevation of medical science. To this end it is essential to have a cordial feeling among the medical men of all sections. The vanquished are apt to feel sore over their defeat, and hence it is commonly easier for the victors to make the first overtures of friendship. We trust that no efforts will be spared to make medical men forget past divisions, and, looking only to the future, go on hand-in-hand to victories greater than those of war, and better for the welfare of the human race. The consolidation of the union of the States ensures the prospect that this nation will, at no remote period, tower among the most gigantic of the earth. It will be our fault if its scientific progress and the ameliorating effects of the healing art do not keep pace with its material grandeur. To this end, the success of the American Medical Association is an important means. It is at present the sole medium of general consultation and intercourse, and our representative before the world. It furnishes an opportunity for men from every part of the land to meet their fellows, compare their ideas, imbibe the grateful sense of unity and fellowship, and to return with enlarged views to distribute a like influence in their own sections. At the time of writing these thoughts, the place of the next meeting has not been determined, but it is not improbable that it may be in some of the Southern cities. If this should be the case, let us see to it that the representation is full and cordial, and that the objects of the organization be carried out with earnestness and power.

The present meeting was opened in the Representatives' Hall, in the State House, at Boston. The meeting was called to order by Dr. N. S. Davis, the President, and opened by prayer by the Chaplain.

Prof. Bigelow, the Chairman of the Committee of Arrangements, then gave an address of welcome to the delegates from all parts of the Union.

Prof. Davis then delivered his annual address. He commenced by congratulating the Association on the glorious victories which have restored our flag to its supremacy in every part of the land, and given us, at last, a return of our brave soldiers, decorated with wreaths of victory and enshrined in a nation's gratitude. He then passed to a notice of the distinguished dead of the year, alluded to the past history of the Association, followed by a discussion of the best methods of conducting its affairs and correcting certain errors in the management of its meetings. He then closed by an appeal to the members to carry forward the work of the organization to the full attainment of its beneficent objects.

---

Chicago Academy of Sciences.—This enterprising society has undergone, during the past year, a complete reorganization. In its new form, it has raised a fund of $70,000, and has established a large scientific museum, with the best of prospects. Mr. Rob't. Kennicott, the Arctic explorer, has been appointed director of the museum, and has now gone, in the interest of the Academy, on a new zoological exploration to the unknown regions near Behring's Straits. Prof. Stimson, the distinguished zoologist from Washington, is curator and secretary of the institution. A superb volume of transactions is now being published, by the liberality of citizens of Chicago, which will be very handsomely illustrated with colored plates of new species of fossils and animals found in the collection of the Academy.

---

Soldier's Home.—The Soldier's Home of this city is a place provided for the residence and treatment of discharged invalid soldiers. It is beautifully situated in the south part of the city, and directly on the lake-shore. It owns a spacious lot and two good buildings, which are occupied by wounded and sick men from the army. The students of Chicago Medical College witnessed there, during the last session, a number of very important surgical operations. The Home is to receive $25,000 of the proceeds of the great fair now in progress in this city. This money will greatly enlarge its capacity and usefulness.

The Committee on Ophthalmology, (Illinois State Medical Society), has called our attention to the fact, that his remarks upon pressure in conjunctivitis, and the use of muriate of ammonia in iritis, were not included in his report, but were made informally, full credit for their use being accorded to Surgeon J. S. Hildreth, of the U.S. Ophthalmic Hospital, in Chicago.

---

Personal.—Prof. H. A. Johnson, of Chicago Medical College, is now on a visit to Europe, for the combined object of the restoration of his health, and of observation on medical matters in Europe. At last accounts he was at Venice, with greatly improved health.

Prof. Chas. Pope, of St. Louis, is also on a visit to the same region, and was recently in Rome.

---

Compliment to an Army Surgeon.—That gallant soldier, Gen. Sherman, who has done so much for the reëstablishment of our union, and the crushing of rebellion, lately remarked in a conversation:—"On my long march from Atlanta to Savannah, not a single wounded or sick man was left behind. They were all brought through, and the few that died were carefully buried, each with a head-board properly marked, and the place recorded; and this I owe to the superior management of my medical director, Surgeon Moore, a man of the highest excellence and capacity.

---

Parisian Hospitals.—M. Maisoneuve, the distinguished French Surgeon, treats varicose veins exclusively by hypodermic injections of perchloride of iron. Many prominent Parisian surgeons have abandoned the practice, almost entirely, of closing wounds by first intention, believing that in this way there is greater danger of erysipelas and pyæmia, than in wounds left open and cauterized. Prof. Pope says that some of them have almost abandoned the knife, using various ingenious cauteries, ecraseurs, etc., from the same dread of pyæmia, believing that by the use of the knife one patient is inoculated with the secretions of another. All this may doubtless happen, but would it not be better to learn to clean their knives better, and ventilate their hospitals more perfectly, instead of returning to the surgery of the dark ages? Erysipelas and pyæmia can be effectually prevented by only two modes, viz., sufficient ventilation, and avoidance of infection. In the light of modern science, the old hospitals of Paris, as well as of other cities, are radically deficient in the supply and distribution of pure air. The world will yet see an entire overthrow of our old system of hospital construction. The old buildings are mostly incapable of being rectified, and will have to be abandoned and better ones built. When this is done fully, and the patients are properly prepared before operations, and properly managed after them, erysipelas and pyæmia will almost cease to be dangers in surgery.

